# Novel Translational Read-through–Inducing Drugs as a Therapeutic Option for Shwachman-Diamond Syndrome

**DOI:** 10.3390/biomedicines10040886

**Published:** 2022-04-12

**Authors:** Valentino Bezzerri, Laura Lentini, Martina Api, Elena Marinelli Busilacchi, Vincenzo Cavalieri, Antonella Pomilio, Francesca Diomede, Anna Pegoraro, Simone Cesaro, Antonella Poloni, Andrea Pace, Oriana Trubiani, Giuseppe Lippi, Ivana Pibiri, Marco Cipolli

**Affiliations:** 1 Cystic Fibrosis Center of Verona, Azienda Ospedaliera Universitaria Integrata, 37126 Verona, Italy; valentino.bezzerri@univr.it (V.B.); anna.pegoraro@aovr.veneto.it (A.P.); 2 Dipartimento di Scienze e Tecnologie Biologiche, Chimiche e Farmaceutiche (STEBICEF), University of Palermo, 90128 Palermo, Italy; laura.lentini@unipa.it (L.L.); vincenzo.cavalieri@unipa.it (V.C.); andrea.pace@unipa.it (A.P.); ivana.pibiri@unipa.it (I.P.); 3 Cystic Fibrosis Center of Ancona, Azienda Ospedaliero Universitaria Ospedali Riuniti, 60126 Ancona, Italy; martina.api86@gmail.com; 4 Hematology Clinic, Università Politecnica delle Marche, AOU Ospedali Riuniti, 60126 Ancona, Italy; e.busilacchi@staff.univpm.it (E.M.B.); a.poloni@staff.univpm.it (A.P.); 5 Zebrafish Laboratory, Advanced Technologies Network (ATeN) Center, University of Palermo, 90128 Palermo, Italy; 6 Department of Medical, Oral and Biotechnological Sciences, G. D’Annunzio University of Chieti-Pescara, 66100 Chieti, Italy; antonella.pomilio@unich.it; 7 Dipartimento di Tecnologie Innovative in Medicina e Odontoiatria, G. D’Annunzio University of Chieti-Pescara, 66100 Chieti, Italy; francesca.diomede@unich.it (F.D.); oriana.trubiani@unich.it (O.T.); 8 Unit of Pediatric Hematology Oncology, Azienda Ospedaliera Universitaria Integrata, 37126 Verona, Italy; simone.cesaro@aovr.veneto.it; 9 Section of Clinical Biochemistry, University of Verona, 37126 Verona, Italy; giuseppe.lippi@univr.it

**Keywords:** bone marrow failure syndromes, ataluren, neutropenia

## Abstract

Shwachman-Diamond syndrome (SDS) is one of the most commonly inherited bone marrow failure syndromes (IBMFS). In SDS, bone marrow is hypocellular, with marked neutropenia. Moreover, SDS patients have a high risk of developing myelodysplastic syndrome (MDS), which in turn increases the risk of acute myeloid leukemia (AML) from an early age. Most SDS patients are heterozygous for the c.183-184TA>CT (K62X) *SBDS* nonsense mutation. Fortunately, a plethora of translational read-through inducing drugs (TRIDs) have been developed and tested for several rare inherited diseases due to nonsense mutations so far. The authors previously demonstrated that ataluren (PTC124) can restore full-length SBDS protein expression in bone marrow stem cells isolated from SDS patients carrying the nonsense mutation K62X. In this study, the authors evaluated the effect of a panel of ataluren analogues in restoring SBDS protein resynthesis and function both in hematological and non-hematological SDS cells. Besides confirming that ataluren can efficiently induce SBDS protein re-expression in SDS cells, the authors found that another analogue, namely NV848, can restore full-length SBDS protein synthesis as well, showing very low toxicity in zebrafish. Furthermore, NV848 can improve myeloid differentiation in bone marrow hematopoietic progenitors, enhancing neutrophil maturation and reducing the number of dysplastic granulocytes in vitro. Therefore, these findings broaden the possibilities of developing novel therapeutic options in terms of nonsense mutation suppression for SDS. Eventually, this study may act as a proof of concept for the development of similar approaches for other IBMFS caused by nonsense mutations.

## 1. Introduction

Shwachman-Diamond syndrome (SDS) belongs to the inherited bone marrow failure syndromes (IBMFS), a group of hematological disorders characterized by enhanced cancer predisposition. SDS patients exhibit exocrine pancreas insufficiency, short stature, bone malformations, and hematologic alterations [1]. Bone marrow is hypocellular in most cases and the myeloid lineage is severely impaired because the maturation of myeloid progenitors is blocked at the myelocyte-metamyelocyte stage [2]. Neutropenia is hence a predictable consequence. In fact, severe neutropenia is observed in the majority of SDS patients, although occurrence of anemia and thrombocytopenia in SDS has been reported. Importantly, almost 15–20% of patients develop a myelodysplastic syndrome, with high risk of developing acute myeloid leukemia [3,4].

SDS is mainly caused by mutations in the Shwachman-Bodian-Diamond Syndrome (*SBDS*) gene, encoding for a ribosomal protein essential for the correct assembly of the 80S eukaryotic ribosome [5]. Although almost 90% of patients with SDS exhibit biallelic mutations in *SBDS*, other genes have been recently associated with SDS, including *EFL1*, *SRP54,* and *EIF6* [6,7,8]. Since all these genes are involved in ribosome biogenesis, SDS has been classified as a ribosomopathy [9]. Of note, approximately 55% of patients harboring biallelic mutations in *SBDS* carry the nonsense mutation c.183-184TA>CT (K62X) in one allele [9].

Unfortunately, no pharmacologic therapy aimed at reducing the leukemic risk nor treatment to improve hematopoiesis in bone marrow failure has been developed so far. Translational read-through-inducing drugs (TRIDs) are of growing interest as promising approaches to correct the basic defect caused by nonsense mutations. Aminoglycosides were the first molecules recognized as TRIDs. They promote the binding of a near-cognate tRNA to a premature termination codon (PTC), thus avoiding termination of translation due to the recruitment of a Class 1 releasing factor, thus leading to nonsense codon suppression [10,11,12]. Aminoglycosides, such as G418 and gentamicin, can directly interact with the 80S eukaryotic ribosomes, binding both the large and the small subunits, as demonstrated by X-ray crystallography and single-molecule FRET [13]. The chemical structure of a specific aminoglycoside establishes its affinity for the ribosome and influences PTC read-through efficiency.

The authors previously reported that ataluren (also known as PTC124), a TRID already approved by the European Medicines Agency (EMA) for treatment of Duchenne muscular dystrophy [14,15], can restore SBDS protein synthesis in bone marrow (BM) hematopoietic progenitor cells as well as in mesenchymal stromal cells (MSC) isolated from bone marrow biopsies of patients with SDS [16]. In our previous study, the restoration of SBDS protein synthesis induced by ataluren was followed by improvement of myeloid differentiation in BM mononuclear cells (BM-MNC). However, similarly to data emerged from in vitro and in vivo studies on DMD [17], almost 23% of ex vivo treatment failed to improve SBDS protein expression and function [16]. Ataluren efficacy is generally cell type-dependent and influenced by the sequence of the stop codon and its flanking regions [18,19,20]. Although ataluren displayed very promising effects in our cell models, thus justifying its clinical development for SDS treatment, the authors decided to extend the preclinical studies to ataluren analogues, which have recently shown improved read-through capabilities in cellular models of cystic fibrosis (CF) [21,22].

Here, the authors report that NV848, an ataluren analogue [23], can promote SBDS protein resynthesis with an efficacy comparable to ataluren in SDS cells. Additionally, NV848 treatment induced SBDS protein expression in both hematological and nonhematological cells, including primary peripheral blood mononuclear cells (PBMC) and periodontal ligament stem cells (PDLSC). Moreover, similarly to ataluren, NV848 promoted ex vivo myeloid differentiation in BM-MNC. Interestingly, NV848 significantly improved in vitro neutrophilic maturation in BM-MNC isolated from SDS patients. Thus, the present study provides further evidence to the efficacy of TRIDs for the treatment of SDS.

## 2. Materials and Methods

### 2.1. Human Samples

Sixteen SDS patients carrying nonsense mutation c.183–184TA>CT in *SBDS* were recruited during the annual outpatient visit at the Cystic Fibrosis Centers of Verona and Ancona, Italy. Clinical signs and genetics of patients recruited in this study are reported in Supplementary Table S1. Bone marrow biopsies (3 mL) and peripheral blood (5 mL) were obtained during programmed hospitalizations. Samples (3 mL) from healthy bone marrow donors for transplant purposes were used as healthy controls. Informed consent was obtained according to the guidelines of the local hospital ethics committees. Lymphoblastoid cell lines, periodontal ligament stem cells (PDLSC), BM-MNC, and plasma samples from a cohort of 16 patients were collected and cryo-preserved according to the IRB approvals (approval number: 658 CESC; CERM 2018-82), along with informed consents.

### 2.2. Cell Cultures

LCLs from patients carrying the c.183–184TA>CT nonsense mutation were obtained as previously described [24]. LCLs (2 × 10^6^) were incubated in RPMI-1640 (Sigma-Aldrich, St. Louis, MO, USA) medium supplemented with 0.5% fetal bovine serum (FBS) (Sigma, St. Louis, MO, USA) for 24 h, to synchronize cell growth. Cells were subsequently incubated in the presence/absence of 2.5 μM ataluren (Selleck Chemicals, Houston, TX, USA), or increasing concentrations of analogues (1–10 μM each), or vehicle alone (DMSO), for 24 h.

PBMC and BM-MNC were isolated from peripheral blood and BM biopsies, respectively, by density gradient centrifugation generated with Ficoll-Paque Plus (Sigma, St. Louis, MO, USA), following the manufacturer’s protocol. Briefly, plasma was removed by centrifuging peripheral blood and BM biopsies at 980× *g* for 10 min. Pellets were diluted 1:2 with PBS and 8 mL of cell suspension was carefully layered on 4 mL of Ficoll-Paque Plus (Sigma, St. Louis, MO, USA) in a 15 mL conical tube. Samples were centrifuged at 400× *g* for 30 min at 20 °C in a swinging bucket rotor without brake. The cell layer at the interphase was aspirated and washed twice with PBS, then suspended with RPMI-1640 supplemented with 10% FBS (PBMC) or with IMDM supplemented with 10% FBS (BM-MNC).

PDLSC were isolated according to a previously reported protocol [25]. Briefly, periodontal ligament biopsies were obtained from human premolar teeth of healthy volunteers or patients with SDS. All periodontal ligament biopsies were de-identified. The periodontal ligament fragments were obtained from alveolar crest and horizontal fibers of the periodontal ligament using a Gracey’s curette. Periodontal tissue specimens were cut into small fragments and supplemented with MSCBM-CD medium (Lonza, Basel, Switzerland) after washing several times with PBS. The medium was changed twice a week, and cells spontaneously migrating from the explant fragments were collected by trypsinization when they reached 80%.

### 2.3. Zebrafish

Wild-type (AB strain) zebrafish (*Danio rerio*) embryos were obtained from the Zebrafish Laboratory of the Advanced Technologies Network (ATeN) Center, University of Palermo. Developing embryos were placed in 96-well culture plates (each well receiving one embryo), maintained at a temperature of 28 ± 0.5 °C and exposed continuously from 6 to 120 h post-fertilization (hpf) to NV848 dissolved in DMSO at increasing concentrations.

### 2.4. Synthesis of Ataluren Derivatives

Ataluren analogues have been synthesized as previously reported [22,26,27]. All synthesized compounds were purified by column chromatography realized by flash silica gel (Merck, Kenilworth, NJ, USA, 0.040–0.063 mm) and mixtures of petroleum ether (fraction boiling at 40–60 °C) and ethyl acetate as eluents. Samples have been analyzed, in order to assess their purity grade (>95%), by HRMS with 6540 UHD Accurate-Mass Q-TOF LC/MS (Agilent Technologies, Inc., Santa Clara, CA, USA) equipped with a Dual AJS ESI source.

### 2.5. Western Blot Analysis

LCLs, PBMCs, and PDLSC were lysed with ice-cold RIPA buffer (1% NP40, 0.5% Na deoxycholate, 0.1% sodium dodecyl sulfate, 150 mM NaCl, 50 mM Tris-HCl pH 8.0), supplemented with 0.25 nM phenylmethanesulfonyl fluoride (PMSF, Sigma, St. Louis, MO, USA) and Complete Protease inhibitor cocktail (Roche, Basel, Switzerland). Protein concentration was determined using the Bradford assay (Bio-rad, Hercules, CA, USA). Forty-five μg of protein per lane was loaded on a 12% sodium dodecyl sulfate polyacrylamide gel (Bio-rad, Hercules, CA, USA). Proteins were transferred to a nitrocellulose membrane (Bio-rad, Hercules, CA, USA) for 5 min at 1.3 A, 25 V in Tris-glycine buffer by Trans-Blot Turbo Transfer System (Bio-rad, Hercules, CA, USA). Membranes were then blocked for 1.5 h with 5% bovine serum albumin (BSA, Sigma, St. Louis, MO, USA) in PBS-Tween-20 (Sigma, St. Louis, MO, USA) at room temperature and incubated overnight with an anti-human SBDS rabbit polyclonal IgG antibody (ab128946, Abcam, Cambridge, MA, USA, dilution 1:300) at 4 °C. The secondary mouse anti-rabbit HRP-conjugated antibody was added at 1:2000 (Sc2357, Santa Cruz Biotechnology, Dallas, TX, USA) in 5% BSA/PBS-Tween. The anti-human HRP-conjugated β-actin mouse monoclonal antibody (A3854, Sigma, St. Louis, MO, USA) was added at 1:5000 in 5% BSA/PBS-Tween-20 and incubated for 1 h at room temperature. Proteins were detected by Clarity Western ECL Substrate (Bio-rad, Hercules, CA, USA). Densitometry was performed by Image Studio Lite Ver 5.2 (LI-COR, Lincoln, NE, USA).

### 2.6. In Vitro Neutrophil Maturation Assay

The immunophenotype of the BM myeloid compartment upon exposure to ataluren or analogues was evaluated by flow cytometry as previously reported [2]. BM samples were depleted of red blood cells by osmotic lysis using the Red Blood Cell Lysis Buffer (Roche, Basel, Switzerland), following the manufacturer’s protocol, and then incubated in IMDM medium (ThermoFisher Scientific, Waltham, MA, USA), supplemented with 20 ng/mL G-CSF (Filgrastim, Hospira, Lake Forest, IL, USA). Cells were incubated with ataluren, or analogues, or vehicle alone (DMSO) for 24 h, then the expression level of CD13, CD33, CD34, CD45, and HLA-DR was evaluated. The possible abnormal expression of mature cell markers (CD11b, CD15) [28] was checked as well. Flow cytometry analysis was performed using a FACSCanto II flow cytometer (BD Biosciences, Franklin Lakes, NJ, USA).

### 2.7. Colony Assays

MNCs were seeded at a density of 10^5^/mL in 6-well plates, in StemMACS HSC-CFU lite medium containing 3 U/mL erythropoietin, supplemented with 20 ng/mL G-CSF (Filgrastim, Hospira, Lake Forest, IL, USA) and supplemented with DMSO vehicle or 2.5 μM ataluren or 10 μM analogues. Granulocyte–macrophage colony forming units (CFU-GM) and burst forming unit-erythroid (BFU-E) were counted every 7 days up to 21 days of incubation using a stereomicroscope.

### 2.8. Orange Acridine Assay

Zebrafish larvae, exposed to ataluren or its analogues, were incubated in E3 medium containing 2.5 µg/mL of AO for 20 min at room temperature and washed in fresh E3 medium 3 times. Before fluorescence stereomicroscope examination, the larvae were anesthetized with 0.03% MS-222 for 3 min.

### 2.9. Locomotor Behavior Assay

The locomotor behavior of untreated, DMSO-, and NV848-treated larvae was assessed at 119 hpf. A 96-well plate containing larvae was transferred into the Zebralab platform (ViewPoint Behavior Technology, Lyon, France), and larvae were allowed to acclimate for 15 min with illumination of the testing chamber set at 50 l×. The activity levels of larvae were then immediately analyzed for 15 min in the same illumination condition. This analysis was performed three times, at the same time of the day, using independent batches of larvae.

## 3. Results

### 3.1. Screening of a Panel of Ataluren Derivatives with Established Readthrough Efficacy

**Figure 1:**
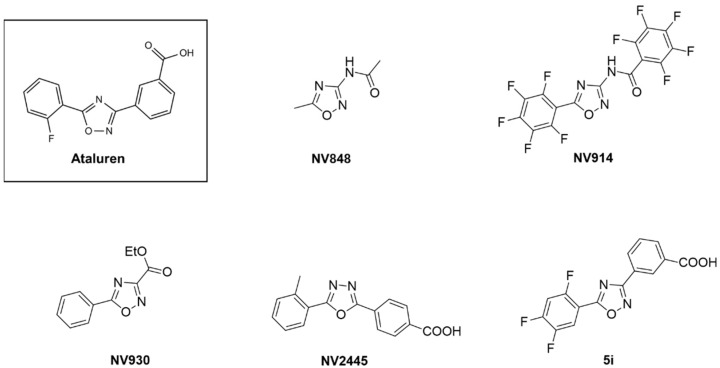
Chemical structures of ataluren derivatives.

Ataluren is a well-known TRID, already approved by EMA for the treatment of DMD [15]. The read-through activity induced by ataluren has been reported in several studies [17,20,29,30,31,32]. Recently, the precise mechanism of action of ataluren in inducing PTC read-through has been described [33]. It has been indeed observed that ataluren promotes PTC read-through by inhibiting release factor-dependent termination of protein synthesis in ribosomes, through orthogonal mechanisms. In addition, in the last decade, ataluren has been pre-clinically and clinically tested in several diseases, including CF [21,34], DMD [14,17,29,35], aniridia [30,36], ciliopathies [37], and SDS [16]. Given the possible limitations of ataluren, including its cell type-dependent efficacy and the competition with other aminoglycosides, the authors recently developed a panel of ataluren analogues (Figure 1). The resulting modified structures induce different electron distribution and distinct properties within the analogues. In fact, the lipophilic carboxyethylic moiety of NV930 could positively influence its absorption process [23]. In the polyfluorinated derivative NV914, the overall polarization of aromatic rings should affect the stacking interactions involved with the biological target; moreover, it improves the molecule’s lipophilicity that is strictly connected to its pharmacokinetics, similarly to, and even more than, 5i [22]. Conversely, the reduced aromaticity of NV848, improving its water solubility, should improve both absorption and biodistribution [23]. Finally, for NV2445, the main structural element of diversity with the others, is the 1,3,4-oxadiazole as a core; this characteristic compared to 1,2,4-oxadiazoles, regardless of substitution patterns, has shown a systematic trend in its pharmaceutical profile. In fact, the 1,3,4-oxadiazole isomer shows an order of magnitude in lower lipophilicity, increased aqueous solubility, and improved metabolic stability [38].

**Figure 2:**
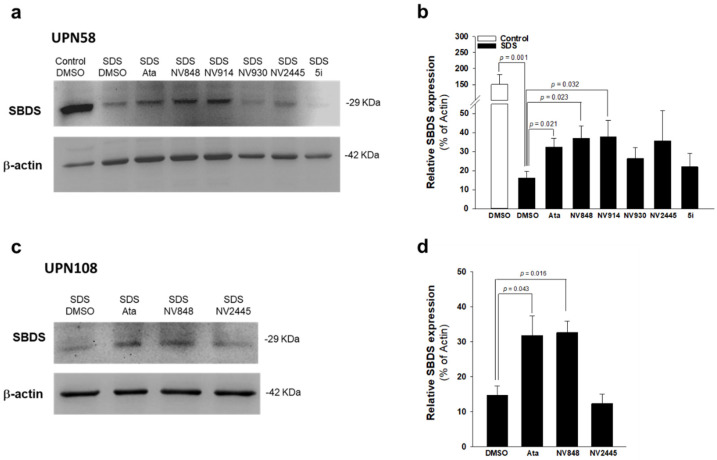
NV848 restores SBDS protein expression in lymphoblastoid cell lines from SDS patients. (**a**), representative experiment; LCL from UPN58 or from healthy donors (Control) were incubated with ataluren (2.5 μM) or analogues (10 μM each), or vehicle alone (DMSO) for 24 h. SBDS protein expression was quantified by Western blot analysis. (**b**), Densitometry analysis of bands as shown in panel (**a**). Data are mean ± SEM of five independent experiments conducted on LCL obtained from patients UPN26, UPN58, UPN75, UPN82, and UPN106. (**c**), PBMC from UPN108 were incubated with ataluren (2.5 μM) or NV848 (10 μM), or vehicle alone (DMSO) for 72 h. SBDS protein expression was quantified by Western blot. (**d**), Densitometry of bands depicted in panel (**c**); data are mean ± SEM of three independent experiments. Student’s *t* test for paired data has been calculated and indicated within the histograms.

Lymphoblastoid cell lines (LCL) were previously generated from B cells isolated from four different SDS patients carrying the nonsense mutation c.183-184TA>CT in SBDS [24]. Cells were incubated with 2.5 μM ataluren, or 10 μM analogues NV848, NV914, NV930, NV2445, and 5i, or vehicle alone (DMSO) for 24 h. These concentrations were previously optimized by other studies conducted both in SDS [16] and CF [22,27,38] cell models. Among the analogues tested, only NV848 and NV914 significantly induced SBDS full-length protein resynthesis, similarly to ataluren, in LCL (Figure 2a,b). Normal LCLs (control) exhibited a 13-fold higher SBDS protein expression compared to SDS cells in the same condition (Figure 2b). Upon treatment with ataluren, NV848, and NV914, SBDS protein synthesis increased by 178%, 250%, and 319%, respectively (Figure 2b). In order to validate the capability of ataluren analogues to restore SBDS protein expression in primary cells, the authors compared the effect of NV848 (more active) with that of NV2445 (less active), on SBDS resynthesis in freshly isolated PBMC. Results confirmed that both 2.5 μM ataluren and 10 μM NV848 can restore full-length SBDS protein expression in primary PBMC (+117% and +123%, respectively) compared with DMSO-treated cells, whereas NV2445 remained ineffective (Figure 2c,d).

### 3.2. Effect of Ataluren Analogues on Myeloid Differentiation of SDS Bone Marrow Hematopoietic Progenitors

**Figure 3:**
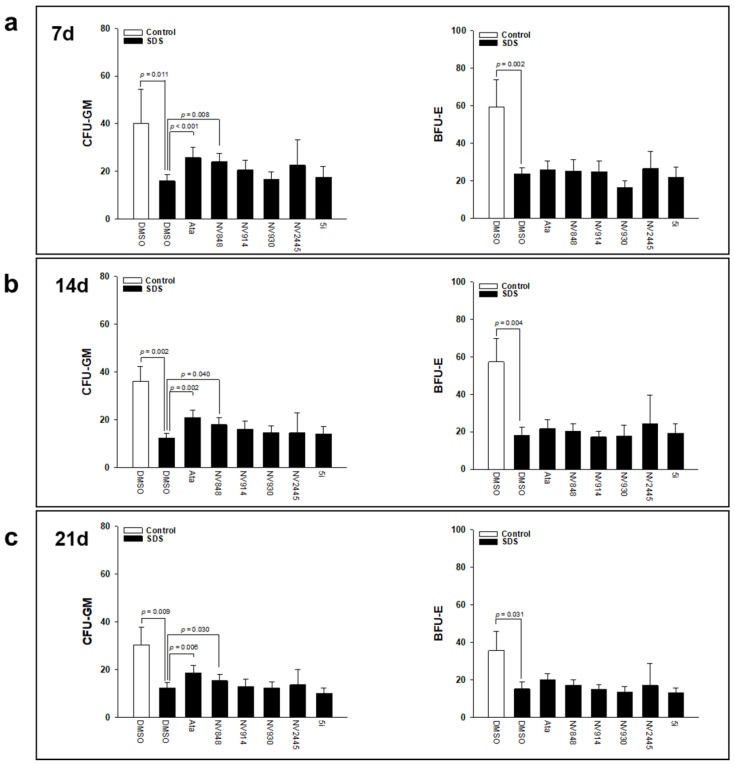
NV848 improves Granulocyte-macrophage colony forming units in BM-MNC isolated from SDS patients. BM-MNC from a cohort of 16 SDS patients were isolated and colony assays were performed. Cells were incubated in the presence of ataluren (Ata, 2.5 μM) or analogues (10 μM each). Granulocyte-macrophage colony forming units (CFU-GM) and burst forming units-erythroid (BFU-E) were counted after 7 days (**a**), 14 days (**b**) and 21 days (**c**) of incubation. Data are mean ± SEM of 16 independent experiments. Student’s *t*-test for paired data has been calculated and indicated within the histograms.

SDS is featured with increased apoptosis of bone marrow CD34^+^ stem cells [39] and the arrest of myeloid progenitors at the myelocyte-metamyelocyte stage [2]. The authors previously reported that ataluren can induce myeloid differentiation of bone marrow hematopoietic progenitors isolated from SDS patients [16]. Here, the authors tested the effect of ataluren analogues on myeloid differentiation ex vivo, in colony assays. BM-MNC isolated from a cohort of 16 patients with SDS carrying nonsense mutations (Supplementary Table S1) were incubated with ataluren or its derivatives for 21 days in a specific semi-solid medium developed for colony assays (Stem-MACS lite medium). Both myeloid and erythroid colonies were counted every seven days. As shown in Figure 3, similarly to ataluren, NV848 almost doubled the number of myeloid colonies after 7 days of treatment (Figure 3a), and at a lesser extent after 14 and 21 days (Figure 3b,c). Although NV914 restored SBDS protein expression in LCL in vitro, it failed to induce myeloid differentiation ex vivo in BM-MNC. Furthermore, both ataluren and NV848 did not improve erythroid differentiation (Figure 3).

### 3.3. NV848 Can Improve Neutrophil Maturation In Vitro, Reducing Expression of Dysplastic Markers in Bone Marrow Hematopoietic Progenitors

**Figure 4:**
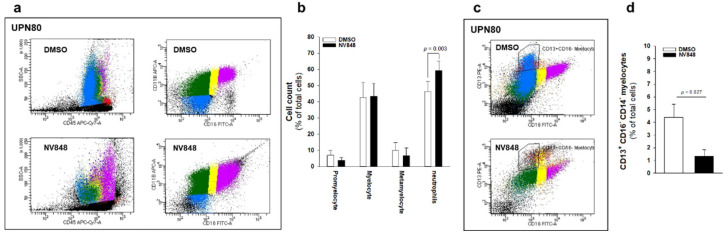
NV848 improves neutrophil maturation in BM-MNC isolated from SDS patients. BM-MNC from a cohort of 6 SDS patients were isolated, and cells were incubated in IMDM medium supplemented with 20 ng/mL G-CSF in the presence of NV848 (10 μM) or vehicle alone (DMSO). (**a**), Representative experiment (UPN80) of neutrophil differentiation, as evaluated by flow cytometry by quantifying the expression of CD16 versus CD11b myeloid markers. Promyelocytes (blue spots), myelocytes (green spots), metamyelocytes (yellow spots), and neutrophils (purple spots) were counted. Red spots represent CD14^+^ cells. (**b**), Histogram representing data collected in panel (**a**); data are Mean ± SEM of six independent experiments conducted on BM-MNC obtained from 6 different SDS patients. (**c**), Representative experiment (UPN80) of neutrophil differentiation, as evaluated by flow cytometry by quantifying the expression of CD16 versus CD13 markers. (**d**), CD13^+^ CD16^−^ CD14^−^ cells (immature neutrophils) were counted in both the experimental conditions described in a; data are mean ± SEM of six independent experiments conducted on BM-MNC obtained from 6 different SDS patients. Student’s *t*-test for paired data has been calculated and indicated within the histograms.

The large majority of SDS patients exhibit severe neutropenia, which in turn leads to increased susceptibility to recurrent infections since the early stages of life. Neutropenia is due to arrest of maturation of myeloid progenitors at the stages of myelocytes and metamyelocytes in bone marrow [2]. Since NV848 induced myeloid differentiation in colony assays, the authors examined its effect on neutrophil maturation in vitro. To this end, the authors incubated BM-MNC isolated from 6 SDS patients with 10 μM NV848 or vehicle (DMSO) for 24 h. Flow cytometric analysis revealed a significant improvement of neutrophil maturation in cells exposed to NV848 (+27.7%), as indicated by CD11b and CD16 expression (Figure 4a,b). A common feature of dysplastic neutrophils is diminished granularity, evidenced by reduced flow cytometric SSC values. Figure 4a shows that NV848 increased SSC already after 24 h. Consistent with this, the percentage of immature neutrophil cells exhibiting dysplastic features (CD13^+^, CD14^−^, and CD16^−^ events) [40] was reduced (Figure 4c,d).

### 3.4. NV848 Restores SBDS Protein Expression Also in Primary Non-Hematological Cells

**Figure 5:**
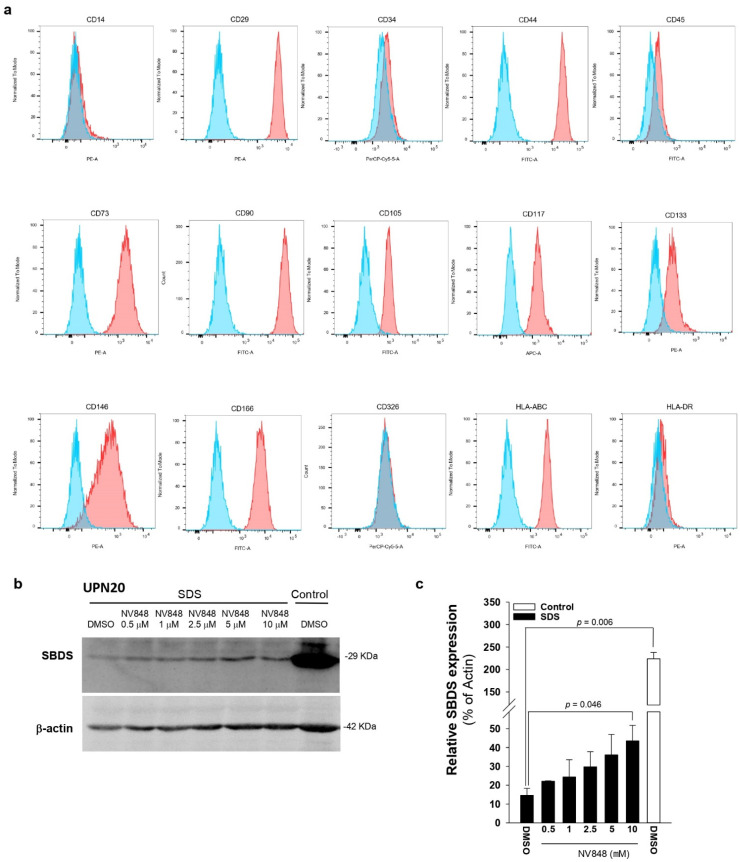
NV848 restores SBDS protein expression in nonhematopoietic PDLSC obtained from SDS patients. (**a**), Immunophenotypic profile of SDS-PDLSC. Cells isolated from the periodontal ligament of patients with SDS were incubated with fluorescent-conjugated antibodies against CD14, CD29, CD34, CD44, CD45, CD73, CD90, CD105, CD117, CD133, CD146, CD166, CD326, HLA-ABC, and HLA-DR, and analyzed by flow cytometry. Red histograms represent cells stained with the relevant antibody; blue histograms show the corresponding IgG isotype control. Data are representative of 4 different samples obtained from 4 patients with SDS. (**b**), PDLSCs from UPN20, or from healthy donor (Control) were incubated with increasing doses (0.5–10 μM) of NV848, or vehicle alone (DMSO) for 24 h. SBDS protein expression was quantified by Western blot analysis. (**c**), Densitometry of bands depicted in panel (**b**). Data are mean ± SEM of 4 independent experiments.

SDS unfortunately lacks exploitable primary cell models for drug development and testing. In previous studies with ataluren, besides LCL, the authors utilized BM hematopoietic stem cells and PBMC [16]. However, these are all hematopoietic models raising the question of TRID efficacy in non-hematopoietic cells. To answer this question, the authors recently isolated stem cells from periodontal ligaments of four SDS patients. As shown in Figure 5a, similarly to PDLSC from healthy individuals, SDS-PDLSCs express a variety of established mesenchymal markers (CD73, CD90, CD105), several surface adhesion molecules (CD29, CD44, CD146, CD166), and a lack of haemopoietic markers (CD14, CD34 and CD45), which confirm their mesenchymal immunophenotype. Healthy PDLSCs express high SBDS protein levels, which are drastically reduced in cells from patients with SDS (Figure 5b,c). When SDS-PDLSC were incubated with increasing concentrations of NV814 for 48–72 h, SBDS levels significantly increased. In particular, 10 μM NV848 enhanced SBDS protein resynthesis by approximately 290% compared with vehicle alone (Figure 5c). These results further confirm, in nonhematological primary cells, that 10 μM is the most effective dose of NV814 for inducing a read-through of PTC, as previously indicated [23].

### 3.5. Toxicity Test in Zebrafish

To assess toxicity, the authors exposed zebrafish embryos to NV848 for a prolonged time. To this end, developing embryos were placed in 96-well culture plates and exposed continuously from 6 to 120 h post-fertilization (hpf) to NV848 at increasing concentrations.

**Figure 6:**
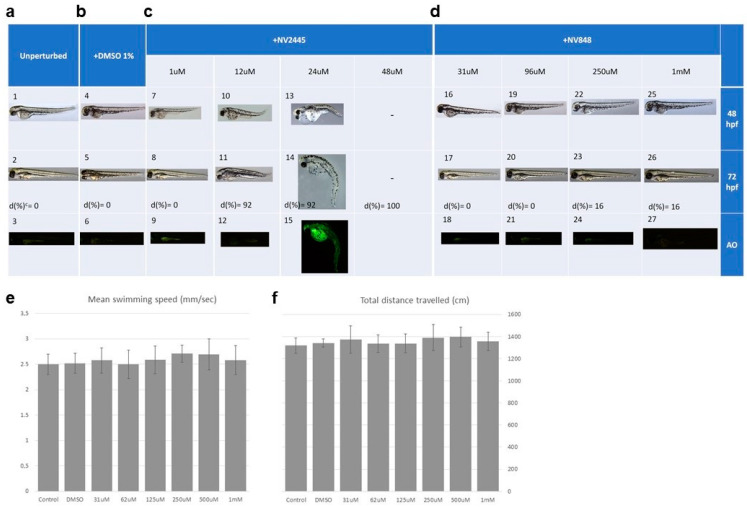
Evaluation of the toxic effects in zebrafish embryos. Toxicity assay and apoptosis assay after exposure to the indicated molecules (**a**): unperturbed control; (**b**): DMSO control; (**c**): NV2445; (**d**): NV848 at the indicated developmental stages. The locomotor behavior of zebrafish embryos after treatment with NV848-Control NV848-treated larvae was assessed at 5 dpf. A 96-well plate containing larvae was transferred into the Zebralab platform and larvae allowed to acclimate for 15 min with the illumination of the testing chamber set at 50 1×. (**e**), Zebrafish swimming velocity upon NV848 treatment compared to control. (**f**), Total distance travelled by zebrafish upon NV848 treatment compared to control. AO, acridine orange staining; d(%), mortality rate.

Twelve replicates were run for each concentration, with each replicate consisting of a single well containing 200 µL of the respective treatment solution and a viable developing embryo, and each experiment was repeated 3 times with independent batches of embryos. Control groups for these experiments included unperturbed embryos and embryos that received 1% DMSO, respectively. Stereomicroscope examination at 48 and 72 hpf revealed that NV848-exposed embryos were morphologically indistinguishable from control unperturbed and DMSO-treated larvae at the same developmental stages (Figure 6a–d).

By contrast, NV2445 caused developmental aberrations in all the observed specimens at concentrations ≥12 µM (Figure 6c). The observed malformations included spinal curvature, pericardial edema, and undetached tail. The mortality rates evaluated at 110 hpf were 0% for unperturbed control and DMSO-treated embryos. Similar or slightly higher values were observed with NV848-treated larvae (0–16%), while exposure to NV2445 at concentrations ≥12 µM frequently caused the death of the larvae with percentages ranging between 92–100% (Figure 6a–d).

Embryo cell apoptosis was assessed at 72 hpf using acridine orange (AO) staining. No obvious apoptotic signals were observed in larvae from control unperturbed, DMSO-treated or NV848-exposed groups (31 μM, 96 μM, 250 μM and 1 mM), except for a characteristic focal point of apoptosis found at the olfactory epithelial cells, which is typically associated with the larval stage examined [41]. By contrast, considerable numbers of apoptotic cells appeared throughout the body of larvae exposed to 24 µM NV2445 (Figure 6c).

Finally, the locomotor behavior was evaluated as a parameter of correct development. The NV848-treated larvae were analyzed at 119 hpf and the activity levels of the larvae were recorded for 15 min with identical illumination condition. The authors found that, compared with control groups, NV848 treatment (31 µM–1 mM) did not affect swimming velocity and total distance travelled by zebrafish larvae throughout the time window examined (Figure 6e,f).

## 4. Discussion

Data emerged from clinical trials showed that chronic ataluren treatment is beneficial to DMD patients undergoing standard care, because it delays the progression of ambulation impairment and the worsening of pulmonary and cardiac functions [42]. Interestingly, clinical studies revealed that the best results are observed in younger individuals, suggesting major benefits of early ataluren administration [43].

However, despite promising pre-clinical results, ataluren failed in clinical studies of CF. In this regard, it should be noted that Phase II trials showed beneficial effects in terms of improvement in total chloride transport, measured by nasal potential difference, in most of the patients enrolled [44,45]. In particular, upon administration of high doses of ataluren, almost 50% of patients exhibited normalized total chloride transport, with increased apical cystic fibrosis transmembrane conductance regulator (CFTR) protein expression on the nasal epithelia [45]. Nevertheless, a first Phase III clinical trial conducted on 238 patients with CF failed its primary outcome, namely the change in percentage, predicted Forced Expiratory Volume in the first second (FEV1) [34]. Contrary to what was observed in Phase II studies, this clinical trial showed no significant difference in total chloride transport. Although a post-hoc analysis revealed that a subgroup of patients not receiving tobramycin were more responsive to ataluren, a second Phase III study involving 279 CF patients in absence of tobramycin therapy failed in reaching both primary and secondary endpoints [21]. The clinical development of ataluren for CF was therefore discontinued. These premises highlight that ataluren clinical benefit in people with CF may be highly variable. Consistent with this, also approximately 39% of patients with DMD did not exhibit protein resynthesis after treatment in a Phase 2a clinical trial [17].

On the basis of shared pathogenetic mechanisms with DMD and selected *CFTR* mutations, the authors exported ataluren into SDS, observing that this drug may enhance SBDS protein expression in SDS cells carrying the nonsense mutation c.183-184TA>CT in the *SBDS* gene [16]. Here, the authors tested the efficacy of analogues NV848, NV914, NV930, NV2445, and 5i on the restoration of SBDS protein expression in SDS cell models, in order to improve the clinical performance of this class of drugs. Second-generation analogues NV848, NV914, and NV930 were optimized from the lead compound NV2445 [38], which showed promising results in correcting nonsense mutated *CFTR* expression in vitro, whereas the 5i compound is a fluoroarylated 1,2,4-oxadiazole molecule, directly derived from ataluren [22]. Similar to ataluren, NV848, NV914, and NV930 have shown to improve CFTR protein resynthesis in fisher rat thyroid cells carrying G542X and W1282X *CFTR* mutations [23]. Interestingly, both mutations generate a PTC with UGA as a stop codon sequence. The UGA stop codon, which represents the best target for ataluren, is present in K62X (c.183-184TA>CT) mutated *SBDS* [19], suggesting that NV2445 derivatives could also be good candidates for suppression of *SBDS* premature stop codon. However, in our SDS cellular models (LCL and PDLSC) both the lead compound NV2445 and its derivative NV930 did not activate a PTC read-through of *SBDS*. Lack of NV2445 and NV930 activity in SDS models is not surprising, because TRID efficacy may vary depending on the type of tissue employed [46] as well as on the biochemical structure of the mRNA sequence harboring the PTC, in particular the sequences of the PTC flanking regions [19,47]. On the contrary, modified NV848 and NV914 significantly induced SBDS protein resynthesis in LCL and primary PBMC from SDS patients. In addition, NV848 significantly improved ex vivo myeloid differentiation of freshly isolated BM hematopoietic stem cells at a similar extent as ataluren. No effect was observed on erythroid differentiation treating BM hematopoietic progenitors with ataluren, nor analogue NV848 instead. These results are in line with our previously reported preclinical study conducted on ataluren in SDS primary cells [16]. The lack of erythroid maturation might be linked to the cell type-dependent efficacy of this class of compounds, which has been already described for ataluren [46]. Interestingly, NV848 induced granulocyte maturation accompanied by a reduced number of CD13^+^ CD16^−^ immature myelocytes and an upward shift of SSC. Interestingly, increased CD13 expression, together with decreased CD16 expression and reduced SSC (i.e., sign of hypogranularity), are well-established features of dysplastic neutrophils [48,49] frequently observed in MDS. These data therefore support the hypothesis that a partial improvement of SBDS resynthesis might reduce, or at least delay, the onset of MDS. Furthermore, NV848 promoted SBDS full-length protein expression in primary mesenchymal PDLSC, suggesting that this type of treatment could be beneficial also in nonhematopoietic tissues.

Overall, in our in vitro/ex vivo tests, the efficacy of NV848 was comparable to that of ataluren. However, this second-generation TRID may have some advantages over ataluren, since it is quite hydrophilic, thus facilitating the selection of administration routes, and did not show any appreciable toxicity up to 1mM concentration in zebrafish experiments. Most importantly, NV848 has shown to be the best candidate for further development in SDS therapy, due to superior absorption, distribution, metabolism, and excretion (ADME) properties, compared to Ataluren and other analogues, as reported [50]. Moreover, this approach could be applied to other IBMFS caused by nonsense mutations, including Diamond-Blackfan anemia, Fanconi anemia, and Dyskeratosis congenital.

## 5. Patents

V.B. and M.C. are co-inventors of the patent WO2018/050706 A1 Method of treatment of Shwachman-Diamond syndrome. L.L., I.P. and A.P. (Andrea Pace) are co-inventors of the patent WO2019101709 “Oxadiazole derivatives for the treatment of genetic diseases due to nonsense mutations”.

## Data Availability

Data supporting the findings of this study are available from the corresponding author upon request.

## Supplementary Materials

The following supporting information can be downloaded at: https://www.mdpi.com/article/10.3390/biomedicines10040886/s1, Table S1: Genetics and clinical features of SDS patients enrolled in this study.
