# Supplementary material for: Novel Translational Read-through–Inducing Drugs as a Therapeutic Option for Shwachman-Diamond Syndrome
Source: Biomedicines. 2022 Apr 12;10(4):886. doi: 10.3390/biomedicines10040886 (PMC9024944; doi:10.3390/biomedicines10040886)
Supplement: Supplementary file 1 [file biomedicines-10-00886-s001.zip › biomedicines-1649223-supplementary.pdf]

Supplementary material

Table S1. Genetics and clinical features of SDS patients enrolled in this study.

**UPN**	Gender	Age	Genotype	PMN (Cell/mm^3)	Phenotype	Cytogenetics
**20**	M	46	258+2T>C/183-184TA>CT	500	PI, FTT, bone malformation, thrombocytopenia	46,XY, del(20)q
**26**	M	16	258+2T>C/183-184TA>CT	58	PI, FTT, bone malformation, thrombocytopenia	46,XY
**37**	F	10	258+2T>C/183-184TA>CT	1280	PI, FTT, recurrent infections	46,XX i(7)(q10)
**39**	M	41	258+2T>C/183-184TA>CT	1300	PI, FTT, bone malformation, thrombocytopenia, cognitive impairment	46,XY
**47**	M	12	258+2T>C/183-184TA>CT	550	PI, FTT, recurrent infections, bone malformation, thrombocytopenia	46,XY
**58**	M	12	258+2T>C/183-184TA>CT	390	PI, FTT, HbF>2%, bone malformation, thrombocytopenia, anemia	46,XY
**69**	F	8	258+2T>C/183-184TA>CT	770	PI, FTT, HbF>2%, anemia, cognitive impairment	46,XX
**72**	M	28	258+2T>C/183-184TA>CT	380	PI, FTT, recurrent infections, HbF>2%, bone malformation, thrombocytopenia, anemia, cognitive impairment	46,XY
**73**	F	7	258+2T>C/183-184TA>CT	520	PI, FTT, HbF>2%, thrombocytopenia	46,XX
**74**	M	9	258+2T>C/183-184TA>CT	1430	PI, FTT, HbF>2%, cognitive impairment	46,XY
**75**	F	7	258+2T>C/183-184TA>CT	1000	PI, FTT, HbF>2%, bone malformation, thrombocytopenia, cognitive impairment	46,XX
**82**	M	8	258+2T>C/183-184TA>CT	310	PI, FTT, recurrent infections, bone malformation, thrombocytopenia, anemia, cognitive impairment	46,XY
**80**	M	7	258+2T>C/183-184TA>CT	680	PI, FTT, recurrent infections, bone malformation, anemia, cognitive impairment	46,XY
**87**	M	18	258+2T>C/183-184TA>CT	880	PI, FTT, recurrent infections, bone malformation, cognitive impairment	46,XY
**106**	M	36	258+2T>C/183-184TA>CT	1210	PI, FTT, bone malformation, anemia	46,XY
**108**	M	17	258+2T>C/183-184TA>CT	970	PS, FTT, bone malformation, anemia	46,XY

UPN, unique patient number; PI, pancreas insufficiency; PS, pancreas sufficiency; FTT, failure to thrive.
